# *Terminalia catappa* attenuates urokinase-type plasminogen activator expression through Erk pathways in Hepatocellular carcinoma

**DOI:** 10.1186/1472-6882-14-141

**Published:** 2014-04-30

**Authors:** Chao-Bin Yeh, Yung-Luen Yu, Chiao-Wen Lin, Hui-Ling Chiou, Ming-Ju Hsieh, Shun-Fa Yang

**Affiliations:** 1 School of Medicine, Chung Shan Medical University, Taichung, Taiwan; 2 Department of Emergency Medicine, Chung Shan Medical University Hospital, Taichung, Taiwan; 3 Graduate Institute of Cancer Biology and Center for Molecular Medicine, China Medical University, Taichung, Taiwan; 4 Department of Biotechnology, Asia University, Taichung, Taiwan; 5 Institute of Oral Sciences, Chung Shan Medical University, Taichung, Taiwan; 6 School of Medical Laboratory and Biotechnology, Chung Shan Medical University, Taichung, Taiwan; 7 Cancer Research Center, Changhua Christian Hospital, Changhua, Taiwan; 8 Institute of Medicine, Chung Shan Medical University, 110 Section 1, Chien-Kuo N. Road, South District, Taichung, Taiwan; 9 Department of Medical Research, Chung Shan Medical University Hospital, Taichung, Taiwan

**Keywords:** Hepatocellular carcinoma, Terminalia catappa, Migration, Invasion, u-PA

## Abstract

**Background:**

The survival rate of malignant tumors, and especially hepatocellular carcinoma (HCC), has not improved primarily because of uncontrolled metastasis. In our previous studies, we have reported that *Terminalia catappa* leaf extract (TCE) exerts antimetastasis effects on HCC cells. However, the molecular mechanisms of urokinase-type plasminogen activator (u-PA) in HCC metastasis have not been thoroughly investigated, and remain poorly understood.

**Methods:**

The activities and protein levels of u-PA were determined by casein zymography and western blotting. Transcriptional levels of u-PA were detected by real-time PCR and promoter assays.

**Results:**

We found that treatment of Huh7 cells with TCE significantly reduced the activities, protein levels and mRNA levels of u-PA. A chromatin immunoprecipitation (ChIP) assay showed that TCE inhibited the transcription protein of nuclear factors SP-1 and NF-κB. TCE also did inhibit the effects of u-PA by reducing the phosphorylation of ERK1/2 pathway.

**Conclusions:**

These results show that u-PA expression may be a potent therapeutic target in the TCE-mediated suppression of HCC metastasis.

## Background

Hepatocellular carcinoma (HCC) is a common malignant neoplasm in Asian countries, and is the fifth most common cancer and the third most common cause of cancer mortality worldwide [1,2]. The survival rate of HCC in Taiwan has not increased primarily because of undamped tumor invasion and metastasis [3,4]. Cancer cells from the primary tumor invade neighboring tissue through the secretion of urokinase-type plasminogen activator (u-PA). This degrades the basement membrane and separates the intercellular matrix, penetrating the circulation system and causing cancer cell invasion and metastasis [5].

The u-PA serine protease is secreted as a 53 KD zymogen (pro-urokinase), and has 3 structural domains: the growth factor domain, kringle domain and serine protease domain [6-8]. The u-PA system includes 4 components: u-PA, urokinase plasminogen activator receptor (u-PAR), and plasminogen activator inhibitors (PAI)-1 and PAI-2. Elevated expression levels of u-PA are correlated with tumor malignancy [5,9]. In general, u-PA is involved in extracellular matrix (ECM) degradation and the invasion and metastasis of cancer cells by regulating the plasminogen/plasmin system. Moreover, the u-PA bound to u-PAR enhances its blood plasminogen activation and capability. This activity is a negative regulator of PAI-1 and PAI-2. Therefore, an imbalance between the u-PA and PAIs contributes to the degradation or deposition of ECM [10,11]. Thus, anti-migration or anti-invasion caused by a u-PA imbalance with mediated suppression may be a way to prevent cancer metastasis. Previous research has shown that u-PA also influences the development of inflammatory, immune, coagulation and fibrinolytic responses [12]. In the metastasis process, u-PA initiates the activation of ECM-degrading enzymes, allowing tumor cells to invade the basement membrane and enter circulation [5,11,13]. Therefore, inhibiting the expression of u-PA can potentially be used to treat cancer metastasis.

Terminalia is a genus of large trees of the flowering plant family combretaceae. This genus includes approximately 100 species distributed in tropical and subtropical regions of Asia, Africa, and Australia. The leaves, bark, and fruit, of *Terminalia catappa* have been used as a folk medicine in several Asian countries for the treatment of dermatitis and as antipyretics. In addition, researchers have identified anticancer compounds in TCE extract, including some flavonoids and hydrolyzable tannins such as punicalagin, punicalin, etc. Therefore, TCE has antioxidative, anti-inflammatory, anti-carcinogenesis, and hepatoprotective effects [14-16]. Our previous study has shown that TCE inhibits MMP-9 expression in HCC cells, producing an anti-invasion and anti-migration effect [3]. However, the molecular mechanisms of u-PA in HCC metastasis remain unclear. Researchers are increasingly focusing on u-PA-mediated cancer metastasis [17-21]. For example, Lu et al. reported that ILF3 promotes breast tumorigenicity by maintaining sustained u-PA expression in breast cancer cells [17]. He et al. showed that DJ-1 was correlated with tumor invasion and worse outcome in pancreatic ductal adenocarcinoma [18]. They also found that these effects were associated with the SRC/extracellular signal-regulated kinase (ERK)/u-PA pathway [18]. Moreover, the transcription of the u-PA gene is regulated by upstream sequences, including motifs corresponding to SP-1 and NF-κB binding sites which act independently or coordinately to regulate u-PA expression [22].

In clinical, other authors have shown that the deregulation of u-PA- and PAI-1-related signaling in metastases might be relevant to lymph node–negative breast cancer tissues [19]. Although only a few studies have investigated the correlation between u-PA and HCC, Itoh et al. reported that u-PA activity may be the most sensitive factor affecting HCC invasion, and that this activity may be a strong predictor of the recurrence of HCC [20]. Zheng et al. showed that u-PA, u-PAR, and PAI-1 may be related to the invasion, metastasis, and prognosis of HCC [21]. Moreover, the u-PA targeting by molecular technologies (ex: antisense RNA or shRNA) [23,24], also in human HCC xenografts decreases the aggressive behavior of HCC cells inhibiting their migration and invasion capabilities [25]. In this study, we attempted to verify the u-PA molecular pathway of TCE in the TCE-mediated suppression of HCC metastasis.

## Methods

### Preparation of Terminalia catappa leaf extracts (TCE)

*Terminalia catappa* leaves were purchased from local herb stores in Taichung, Taiwan. The *Terminalia catappa* ethanol extract (TCE) used in this study was prepared by an initial condensation followed by lyophilization, as described previously [26]. The plant material was identified at the Department of Biochemistry, Chung Shan Medical University in Taichung. A voucher specimen is deposited by Dr. Yih-Shou Hsieh. Briefly, 100 g of air-dried leaves were boiled at 70°C for 24 h in 500 mL of 50% ethanol. The extraction procedure was repeated twice. The solvent was then removed from the combined extract with a vacuum rotary evaporator. The filtrate was then lyophilized and stored at -20°C. The chemical profile of the TCE was analyzed using high-pressure liquid chromatography (HPLC)–mass spectrometer, as described previously [26]. For subsequent experiments, the dried TCE powder was dissolved in 50% dimethyl sulfoxide (DMSO) to achieve an indicated concentration with the highest concentration of DMSO being less than 0.1%.

### Cell culture and TCE treatment

HCC (Huh7) cells obtained from Food Industry Research and Development Institute (Hsinchu, Taiwan) were cultured in Dulbecco’s modified Eagle’s medium (Life Technologies, Grand Island, NY, USA), 10% fetal bovine serum, 2 mM glutamine, 100 U/mL penicillin, 100 μg/mL streptomycin, and 400 ng/mL hydrocortisone. All cell cultures were maintained at 37°C in a humidified atmosphere of 5% CO2. For TCE treatment, appropriate amounts of stock solution of TCE were added to the culture medium to achieve the indicated concentrations. The cells were then incubated for indicated time periods, and dimethyl sulfoxide solution without TCE was used as a blank reagent.

### In vitro wound closure

Huh7 cells (1 × 10^5^ cells/well) were plated in 6-well plates for 24 h, and then wounded by scratching with a pipette tip. The injured cells were then incubated with DMEM medium containing 0.5% FBS and treated with or without TCE (0, 25, 50, 75, and 100 μg/mL) for 0, 12, or 24 h. The cells were photographed using a phase-contrast microscope (×100).

### Cell invasion and migration assays

Cell invasion and migration were assayed following the methods described by Yang et al. [27]. After treatment with TCE (0, 25, 50, 75, and 100 μg/mL) for 24 h, the surviving cells were harvested and seeded in a Boyden chamber (Neuro Probe, Cabin John, MD, USA) at 10^4^ cells/well in serum-free medium and then incubated for 24 h at 37°C. For invasion assay, 10 μL Matrigel (25 mg/50 mL; BD Biosciences, MA, USA) was applied to polycarbonate membrane filters with an 8-μm pore size, and the bottom chamber contained standard medium. Filters were then air-dried for 5 h in a laminar flow hood. The invaded cells were fixed with 100% methanol and stained with 5% Giemsa. Cell numbers were counted under a light microscope. The migration assay was conducted as described in a previous study by an invasion assay with no Matrigel coating [28].

### Determination of u-PA by casein zymography

The activities of u-PA in a serum-free conditional medium were measured using casein zymography protease assays, as described previously [28]. Briefly, prepared samples containing 20 μL conditional medium were loaded onto a precast 8% sodium dodecyl sulfate–polyacrylamide gel containing 2% casein and 20 μg/mL plasminogen. After electrophoresis, the gel samples were washed with 2.5% Triton X-100 and incubated in a reaction buffer (40 mM Tris–HCl, pH 8.0; 10 mM CaCl2; and 0.01% NaN3) for 12 h at 37°C. The gels were then stained with Coomassie brilliant blue R-250.

### RNA preparation and TaqMan quantitative real-time PCR

Total RNA was isolated from HCC cells using Trizol (Life Technologies, Grand Island, NY) following the manufacturer’s instructions. Quantitative real-time PCR analysis was conducted using Taqman one-step PCR Master Mix (Applied Biosystems). A 100-ng sample of cDNA was added to each 25 μl reaction with u-PA or GAPDH primers and TaqMan probes. Quantitative real-time PCR assays were conducted in triplicate on a StepOnePlus sequence detection system. The threshold was set above the non-template control background and within the linear phase of the target gene amplification to calculate the cycle number at which the transcript was detected.

### Preparation of total cell lysates

For total cell lysate preparation, cells were rinsed with PBS twice and scraped with 0.2 mL of cold RIPA buffer containing protease inhibitors cocktail, and then vortexed at 4°C for 10 min. Cell lysates were subjected to centrifugation at 10,000 rpm for 10 min at 4°C, and the insoluble pellet was discarded [29]. The protein concentration of total cell lysates and nuclear fraction were determined by Bradford assay [30].

### Western blot analysis

The samples containing 20 μg cell lysates were separated in a 10% polyacrylamide gel and transferred onto a nitrocellulose membrane. Each blot was subsequently incubated with 5% non-fat milk in Tris-buffered saline (20 mM Tris, 137 mM NaCl, pH 7.6) for 1 h to block non-specific binding. Each blot was then incubated overnight with specific antibodies for u-PA, PAI-1, β-actin, unphosphorylated or phosphorylated activated forms of the corresponding ERK1/2, JNK1/2, p38, and Akt. The blots were then incubated with a horseradish peroxidase goat anti-rabbit or anti-mouse IgG for 1 h. Signals were detected using an enhanced chemiluminescence (ECL) commercial kit (Amersham Biosciences), and the relative photographic density was quantitated by scanning the photographic negatives on a gel documentation and analysis system (AlphaImager 2000, Alpha Innotech Corporation, San Leandro, CA, USA).

### Transfection and u-PA promoter-driven luciferase assays

Huh7 cells were seeded in 6-well cell culture plates at a concentration of 5 × 10^4^ cells per well. After 24 h of incubation, pGL3-basic (vector) and u-PA promoter plasmid were co-transfected with a β-galactosidase expression vector (pCH110) into cells using Turbofect (Fermentas, Carlsbad, CA). After 12 h of transfection, cells were treated with vehicle or TCE (0, 25, 50, 75, and 100 μg/mL) for 24 h. After the cell lysates were harvested, a luciferase assay kit was used to determine the luciferase activity. The value of the luciferase activity was normalized to transfection efficiency and monitored by β-galactosidase expression.

### Chromatin immunoprecipitation analysis (ChIP)

Chromatin immunoprecipitation analysis was performed as described previously [31]. First, DNA immunoprecipitated with anti-NF-κB and anti-SP-1 was purified and extracted using phenol-chloroform. This immunoprecipitated DNA was then analyzed by PCR or quantitative PCR with specific primers.

### Statistical analysis

Data are indicated as mean ± SD of three different determinations. For all of the measurements, analysis of variance followed by Student’s t-test (Sigma-Stat 2.0, Jandel Scientific, San Rafael, CA, USA) was used to assess the differences between control and cells treated with various concentration of TCE. A difference of *P* < 0.05 was considered to be statistically significant and the experiments were repeated 3 times.

## Results

### Effects of TCE on the Protein Levels of u-PA and its Endogenous Inhibitor PAI-1

**Figure 1:**
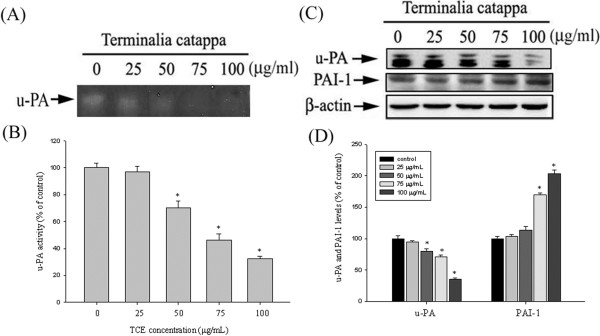
**Effects of TCE on the Protein Levels of u-PA and its Endogenous Inhibitor PAI-1. (A-B)** Huh7 cells were treated with TCE (0–100 μg/mL) for 24 h and then subjected to casein zymography to analyze the activity of u-PA. **(C)** Huh7 cells were treated with TCE (0–100 μg/mL) for 24 h and then subjected to western blotting to determine the protein levels of u-PA and PAI-1. **(D)** Quantitative results of u-PA and PAI-1 protein levels, which were adjusted with the β-actin protein level. The values in this figure represent the means ± SD of at least 3 independent experiments. *p < 0.05 as compared with the vehicle group.

Huh7 cells were treated with TCE (0, 25, 50, 75, and 100 μg/mL) in a serum-free conditional medium for 24 h and then subjected to casein zymography for the analysis of u-PA activity. As Figures 1A and 1B show, TCE treatment may lead to reduced activity of u-PA in a dose-dependent manner. According to western blotting analyzed in the cell lysates, the TCE substantially reduced u-PA protein expression (Figure 1C). This result shows that the migration inhibitory effects of TCE at least partially inhibited u-PA expression. An investigation of the effects of TCE on the protein expression of the u-PA endogenous inhibitor (PAI-1) shows that TCE induced PAI-1 upregulation in a concentration-dependent manner (Figures 1C and 1D).

### Effects of TCE in suppressing u-PA expression at a transcriptional level

**Figure 2:**
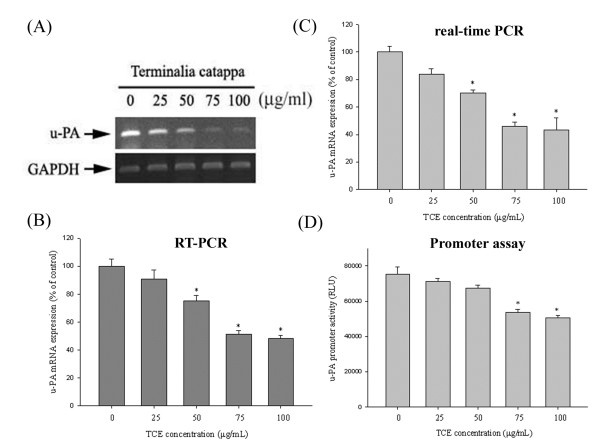
**Effects of TCE suppression on u-PA expression at a transcriptional level.** Huh7 cells were treated with TCE (0, 25, 50, 75, and 100 μg/mL) for 24 h and then subjected to RT-PCR **(A, B)** and quantitative real-time PCR **(C)** to analyze the mRNA expression of u-PA. **(D)** u-PA promoter reporter assay to analyze the promoter activity of u-PA. Luciferase activity, determined in triplicate, was normalized to β-galactosidase activity. The values in this figure represent the means ± SD of at least 3 independent experiments. *p < 0.05 as compared with the vehicle group.

The results of mRNA testing, reverse transcription PCR (RT-PCR), real-time PCR, and promoter reporter assays revealed the inhibitory effects of TCE on u-PA mRNA expression in Huh7 cells. The Huh7 cells in this study were treated with 0, 25, 50, 75, and 100 μg/mL of TCE for 24 h and were then subjected to RT-PCR and real time-PCR to analyze mRNA levels. The u-PA mRNA levels decreased considerably in a concentration-dependent manner after treatment with various concentrations of TCE (Figures 2A- 2C). Figure 2D shows that the luciferase activities of u-PA were significantly suppressed, as determined by a luciferase assay kit. These results show that TCE regulates the expression of u-PA, at least partially, at the transcriptional level.

### The critical role of SP-1 & NF-κB in TCE -induced transcriptional inhibition of u-PA in Huh7 cells

**Figure 3:**
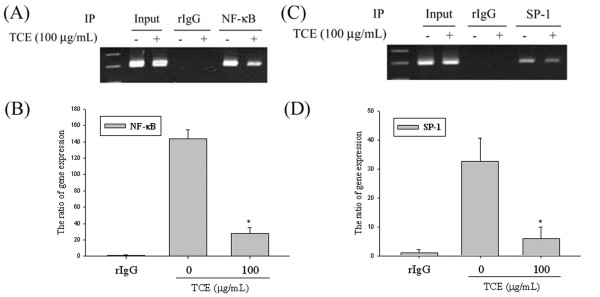
**The critical role of NF-κB and SP-1 in TCE -induced transcriptional inhibition of u-PA in Huh7 cells.** Huh7 cells were treated with TCE 100 μg/mL for 24 h and the nuclear fraction was prepared as described in the Methods Section. This figure shows the ChIP analysis of the association of **(A-B)** NF-κB and **(C-D)** SP-1 transcription factors with the u-PA promoter region in Huh7 cells. The values in this figure represent the means ± SD of at least 3 independent experiments. *p < 0.05 as compared with the vehicle group.

A ChIP assay was also performed to investigate the effects of TCE on NF-κB and SP-1 DNA-binding activities and confirm the transcription factor involvement in the transcriptional inhibitory effects of TCE on u-PA. Figures 3A and 3C show the levels of SP-1 & NF-κB in a nucleus that was immunodetected with NF-κB and SP-1 specific antibodies, respectively. These findings indicate that TCE might induce the transcriptional inhibition of u-PA in Huh7 cells by suppressing NF-κB and SP-1 binding activity (Figures 3B and 3D).

### Effects of TCE on the MAPKs pathway and PI3K/Akt signaling

**Figure 4:**
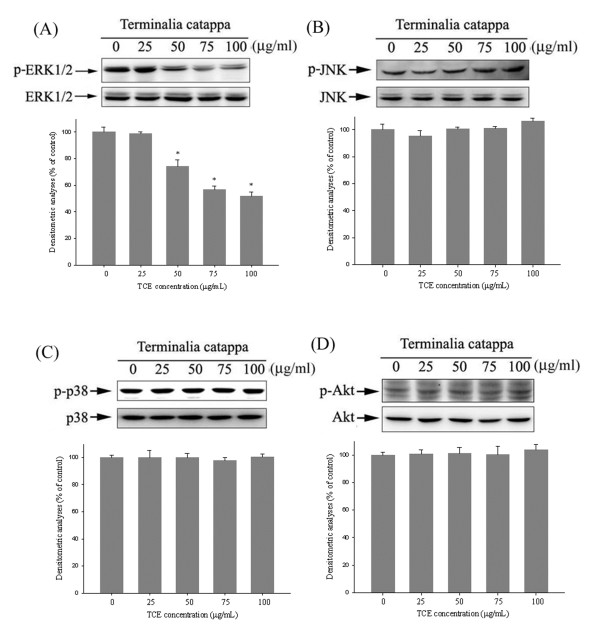
**Effects of TCE on the MAPKs pathway and PI3K/Akt signaling.** Huh7 cells were cultured in various concentrations of TCE (0, 25, 50, 75, and 100 μg/mL) for 24 h. The cell lysates were then subjected to SDS–PAGE followed by western blots with **(A)** anti-ERK1/2, **(B)** anti-JNK, **(C)** anti-p38, and **(D)** anti-Akt (total and phosphorylated) antibodies, as described in the Methods Section. The activities of these proteins were subsequently quantified by densitometric analyses, with the control being 100% (shown just below the gel data). The values in this figure represent the means ± SD of at least 3 independent experiments. *p < 0.05 as compared with the vehicle group.

To further investigate the mechanisms underlying the upstream signaling pathways of u-PA, we used western blotting to evaluate the effects of TCE on the MAPK and Akt pathways. Western blotting showed that TCE could reduce the phosphorylation of ERK 1/2 (Figure 4A) in Huh7 cells. The densitometric analyses of blots compared to the control shows that the treatment of TCE at 50 μg/mL could reduce in phosphorylation of ERK 1/2. However, the phosphorylation of the p38, JNK1/2, and Akt pathways remained unaffected (Figures 4B- 4D). Therefore, we suggest that the activation of the Erk1/2 signaling pathway is required for TCE to suppress u-PA.

### Effects of TCE and ERK1/2 inhibitor (U0126) on u-PA expression, in vitro wound closure, cell migration, and invasion in Huh7 cells

**Figure 5:**
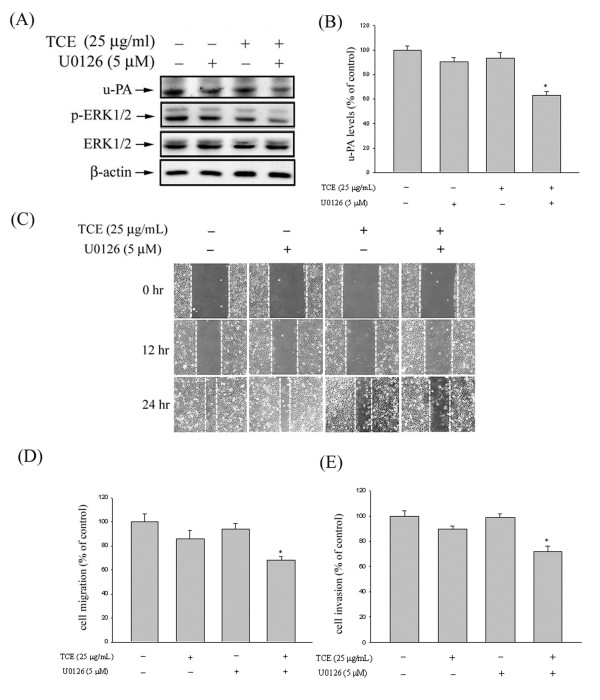
**Effects of TCE and ERK1/2 inhibitor (U0126) on u-PA expression, in vitro wound closure, cell migration, and invasion in Huh7 cells. (A-B)** Huh7 cells were pretreated with U0126 for 30 min and then incubated in the presence or absence of TCE for 24 h. The cell lysates were then subjected to SDS–PAGE followed by western blots with anti-u-PA antibodies, as described in the Methods Section. **(C-E)** Huh7 cells were pretreated with U0126 for 30 min and then incubated in the presence or absence of TCE for 24 h. The Huh7 cells were then subjected to in vitro wound closure, cell migration, and invasion assay. The migration and invasion abilities of Huh7 cells were quantified by counting the number of cells that invaded the underside of the porous polycarbonate, as described in the Methods Section. The values in this figure represent the means ± SD of at least 3 independent experiments. *p < 0.05 as compared with the vehicle group.

To further delineate whether the inhibition of proteinase, invasion, and migration by TCE occurred through the inhibition of the ERK1/2 signaling pathway, we investigated the effects of a specific inhibitor of the ERK1/2 pathway (U0126) on Huh7 cells. These results show that combined treatment of the inhibitor with TCE further decreased u-PA expression (Figures 5A and 5B). We also observed a similar trend in the inhibition of Huh7 migration and invasion with combined treatment (Figures 5C- 5E). Therefore, the inhibition of the ERK1/2 signaling pathways may reduce the expression of u-PA and tumor cell invasion.

## Discussion

Metastasis is the process of a cancer cell breaking away from its original tumor and invading either the circulatory system or lymphatic system, which carries it to a new location, where it positions itself in a new site [32,33]. Although the process of metastasis of cancer cells involves multiple steps and various cytophysiological modifications to remodel itself to another place, ECM degradation or breakdown caused by a protease is the major step in tumor invasion or migration [34]. This phenomenon leads to the disjunction of the intercellular matrix, which promotes cancer cell mobility and eventually leads to metastasis. Among the proteases involved, u-PA plays one of the most important roles in cancer invasion and metastasis [9]. The results of this study confirm that TCE has an inhibitory effect on metastasis through u-PA in HCC cells. This finding is similar to our previous research of the compound norcantharidin and resveratrol, which inhibit u-PA expression to produce anti-migration potential effects on HCC cells [4,35].

Doctors around the world, and especially in Asian countries, have been emphasizing the prevention and treatment of various chronic diseases through the clinical application of traditional Chinese medicine. Previous studies on folk medicine have shown that TCE is an important herbal food drug that has various biological effects, including antioxidant, hepatoprotective, and antimetastasis effects, and the prevention of hepatocyte apoptosis [3,16,36-38]. Our previous study reported that TCE has an inhibitory effect on metastasis in HCC cells [3], but the anti-migration pathway of u-PA on HCC cells remains unclear. This study shows that TCE treatment significantly inhibited the migration and invasion capacities of Huh 7 cell lines with low cytotoxicity in vitro through the down-regulation of metastasis-associated u-PA of the transcription factors SP-1 and NF-κB.

The mitogen-activated protein kinase (MAPK) family of serine/threonine kinases includes Jun-N-terminal kinase (JNK), p38, and ERKs. The mammalian ERK1 and ERK2 (ERK1/2) have more than 80% amino acid identity, and are copiously present in all tissues [14,39,40]. Alterations of the ERK pathway are well documented in human HCC cells. The phosphorylated ERK levels are significantly increased in most human HCC samples [1,41]. The ERK pathway increases the expression levels of matrix metalloproteinases (MMPs), thus facilitating cell migration and tumor cell invasion [1,42,43]. However, research on the MAPK pathway and its u-PA-mediated anti-migration effects on HCC cells are rare. The results of this study, confirm that TCE inhibits the phosphorylation of the ERK1/2 pathway, leading to a down-regulation of metastasis-associated u-PA.

The expression of the u-PA gene is chiefly regulated by activators or inhibitors and cell surface localization at the transcriptional, posttranscriptional, and protein levels. Tang et al. reported that the u-PA system plays an important role in breast cancer growth, invasion, and metastasis, which may work through the Ras-ERK or p38-MAPK pathways [11]. The u-PA gene is also crucial to the choice of adjuvant therapies in node-negative breast cancer because it provides information regarding the relapse risk and treatment response [11]. Furthermore, Huang et al. reported that SDF-1 increases SP-1 DNA-binding activities in colon cancer cell lines. The inhibition of SP-1 activation blocked the SDF-1-induced expression and activity of the u-PA promoter [44]. In this study, we show that TCE inhibits u-PA promoter in Huh7 cells through SP-1 and NF-κB, suppressing cell invasion and metastasis.

For evaluation of the inhibitory effect on the invasiveness and migration of human Hepatocellular carcinoma Huh7 cells by TCE, we chose the concentration range up to 100 μg/mL which had no cytotoxic effect on Huh7 cells and it is consistent with in vitro studies from other laboratories [45]. Moreover, the toxicity of TCE has been reported that an oral administration of 3,000 mg/kg TCE did not cause any lethality in the single-dose acute toxicity test and the treatment by 3,000 mg/kg/day for 30 continuous days did neither alter the body weights nor the hematological parameters in C57BL/6 mice in the study [26]. For the in vivo study, the inhibitory effect of TCE on the growth and metastasis of Lewis lung carcinoma cells (LLC) in vivo was proven in the previously study [26]. Therefore, more animal studies and clinical trials using the concentration range of TCE are needed to further justify its clinical value.

## Conclusion

This study shows that TCE has an inhibitory effect on several crucial steps of metastasis, including cell invasion and migration, by regulating the activities and protein level of u-PA and its natural inhibitor. We also demonstrated that TCE effectively inhibits the phosphorylation of ERK1/2 signaling pathways by downregulating the transcription factors SP-1 and NF-κB DNA binding activities, leading to u-PA suppression and inhibiting metastasis. The signal transduction mediators and transcriptional factors involved in the TCE anti-migration potential effect on the HCC cells may lead to the development of specific mediation to inhibit cell metastasis.

## Abbreviations

TCE: Terminalia catappa leaf extracts; u-PA: urokinase-type plasminogen activator; PAI: Plasminogen activator inhibitors; HCC: Hepatocellular carcinoma; ChIP: Chromatin immunoprecipitation; HPLC: High-pressure liquid chromatography.

## Competing interests

The authors declare that they have no competing interests.

## Authors’ contributions

CBY and SFY conceived and designed the study. MJH and CWL performed the experiments. MJH, YLY, and SFY analyzed the data. CBY and HLC drafted the manuscript. All authors read and approved the final manuscript.

## Pre-publication history

The pre-publication history for this paper can be accessed here:

http://www.biomedcentral.com/1472-6882/14/141/prepub
